# Relationship Between Putative *eps* Genes and Production of Exopolysaccharide in *Lactobacillus casei* LC2W

**DOI:** 10.3389/fmicb.2018.01882

**Published:** 2018-08-17

**Authors:** Xin Song, Zhiqiang Xiong, Linghui Kong, Guangqiang Wang, Lianzhong Ai

**Affiliations:** Shanghai Engineering Research Center of Food Microbiology, School of Medical Instrument and Food Engineering, University of Shanghai for Science and Technology, Shanghai, China

**Keywords:** exopolysaccharide, knockout, overexpression, gene complementation, *Lactobacillus casei*

## Abstract

*Lactobacillus casei* LC2W, a probiotic strain, can produce exopolysaccharide (EPS) with anti-hypertensive bioactivity. The relationship between *eps* genes and EPS synthesis in LC2W due to unclear regulation mechanism of EPS biosynthesis was investigated. The several relevant genes in EPS biosynthetic gene cluster were deleted, overexpressed and complemented. The results suggested that glucose-1-phosphate thymidyltranseferase gene (*LC2W_2179*), uncharacterized EPS biosynthesis protein (*LC2W_2188*), and EPS biosynthesis protein (*LC2W_2189*) were related to EPS biosynthesis. EPS titer decreased 15, 13, and 21% when the three genes were deleted, respectively. When they were overexpressed, EPS titer increased 16, 10, and 18%. When they were complemented, EPS titer was similar to the wild-type strain. This work showed the three *eps* genes from LC2W played important roles on EPS production.

## Introduction

Lactic acid bacteria (LAB) are widespread in natural environment and play an important role in human health. Most of LAB are able to produce exopolysaccharides (EPSs) with a wide structural diversity. These polymers are usually recognized as safe bioactive compounds (Ruas-Madiedo et al., 2006; Mozzi et al., 2009; Mitsuoka, 2014). In recent years, EPSs produced by LAB have received an increasing interest since they might be responsible for several health benefits attributed to probiotic strains, such as immune stimulation, anti-mutagenicity, anti-tumor activity, anti-gastritis, anti-ulcer, anti-virus, cholesterol-lowering properties, blood pressure-lowering activity (Nagaoka et al., 1994; Rodriguez et al., 2009; Kanmani et al., 2011; Fanning et al., 2012; Hidalgo-Cantabrana et al., 2012; Nacher-Vazquez et al., 2015). Moreover, EPS are extensively used in the industrial production of fermented milk and cheese as natural thickeners, stabilizers, emulsifiers, and texturizers (Broadbent et al., 2003; Patel et al., 2012; Ryan et al., 2015). Furthermore, EPSs are also used as food additives and functional food ingredients (Jolly et al., 2002; Welman and Maddox, 2003). However, there is a need to improve the yield of EPSs as well as to explain the biosynthesis mechanism in the LAB.

Over the past few years, EPSs productions from LAB have been improved through mutagenesis breeding and culture conditions optimization (Looijesteijn et al., 2000; Lamothe et al., 2002; Seesuriyachan et al., 2012; Liu et al., 2016). The genes related to EPSs synthesis might be located in plasmids or on the chromosome. In *Lactococcus lactis* NIZO B40, all the essential genes required for biosynthesis of EPS are in a single 12-kb gene cluster located on a 40-kb plasmid (van Kranenburg et al., 1997). In *Streptococcus thermophiles* Sfi6, EPS synthesis is associated with a 15.25-kb region encoding 16 open reading frames (ORFs) located on the chromosome (Stingele et al., 1996). Many studies indicated that the yield of EPSs was improved by overexpressing some specific genes or gene cluster. For example, overexpression of a complete *eps* gene cluster in *L. lactis* was capable of raising EPS production levels (Boels et al., 2003). Welman et al. (2006) suggested that glucose-6-phosphate may be vital in EPS synthesis. van Kranenburg et al. (1999), also indicated that overexpressed glucosyltransferase could increased EPS production by 15%.

*Lactobacillus casei* has been used as a health-promoting microbe in fermented food for centuries. It plays an important role in decreasing inflammation, regulating the intestinal microenvironment, and reducing stress-associated abdominal dysfunction in healthy subjects exposed to stressful situations (Aktas et al., 2016; Gleeson et al., 2016; Kato-Kataoka et al., 2016).

*Lactobacillus casei* LC2W, an EPS-producing strain used in this study, was isolated from traditional dairy products in Inner Mongolia (Ai et al., 2008). Complete genome sequence of LC2W has been finished and a precise effective genetic tool has been proved in *L. casei* (Chen et al., 2011; Song et al., 2017). Gene knockout, overexpression and complementation mutants were constructed and EPS titer of mutants and wide-type strain were evaluated. The relationship between *eps* genes and EPS production was explored in this study.

## Materials and Methods

### Bacterial Strains, Media, and Culture Conditions

*Escherichia coli* DH5α and Top 10 was used as cloning host. It grew on Luria-Bertani broth (LB) medium (1% (W/V) NaCl, 1% (w/v) tryptone, and 0.5% (W/V) yeast extract) with kanamycin (50 mg/L) at 30°C. All overexpression plasmids were introduced in competent *E. coli* Top 10 cells grown in LB medium with erythromycin (400 mg/L). *L. casei* LC2W was cultured in Man, Rogosa, and Sharpe (MRS) medium (each liter of which contained 10.0 g peptone, 10.0 g beef extract, 4.0 g yeast extract, 20.0 g glucose, 1 ml Tween 80, 2.0 g dipotassium hydrogen phosphate, 5.0 g sodium acetate, 2.0 g triammonium citrate, 0.2 g magnesium sulfate, and 0.05 g manganese sulfate) at pH 6.2 and 37°C without shaking (Chen et al., 2014). Erythromycin was used at a concentration of 10 mg/L when necessary. The agar concentration was added at 15 g/L. All recombinant plasmids were introduced into LC2W competent cells by electroporation. The strains used in this study are listed in **Table 1**.

**Table 1:** Plasmids and strains used in this study.

Strains or plasmids	Description	Source or reference
**Strains**		
*L. casei* LC2W	Wild-type	Our lab
*E. coli* DH 5α	Commercial transformation host	GIBCO BRL, Life Technologies
*E. coli* Top 10	Commercial transformation host	Takara (Japan)
LC2WΔ2179	*LC2W_2179* knockout mutant of LC2W	Our lab
LC2WΔ2182	*LC2W_2182* knockout mutant of LC2W	This study
LC2WΔ2187	*LC2W_2187* knockout mutant of LC2W	This study
LC2WΔ2188	*LC2W_2188* knockout mutant of LC2W	This study
LC2WΔ2189	*LC2W_2189* knockout mutant of LC2W	Our lab
LC2W pIB184-2179	*LC2W_2179* overexpression mutant	This study
LC2W pIB184-2182	*LC2W_2182* overexpression mutant	This study
LC2W pIB184-2187	*LC2W_2187* overexpression mutant	This study
LC2W pIB184-2188	*LC2W_2188* overexpression mutant	This study
LC2W pIB184-2189	*LC2W_2189* overexpression mutant	This study
LC2WΔ2179 pIB184-2179	*LC2W_2179* complemented mutant	This study
LC2WΔ2182 pIB184-2182	*LC2W_2182* complemented mutant	This study
LC2WΔ2187 pIB184-2187	*LC2W_2187* complemented mutant	This study
LC2WΔ2188 pIB184-2188	*LC2W_2188* complemented mutant	This study
LC2WΔ2189 pIB184-2189	*LC2W_2189* complemented mutant	This study
**Plasmids**		
pLCNICK	Genetic edit plasmid	Our lab
pLCNICK-2182	Gene knockout plasmid for *LC2W_2182*	This study
pLCNICK-2187	Gene knockout plasmid for *LC2W_2187*	This study
pLCNICK-2188	Gene knockout plasmid for *LC2W_2188*	This study
pIB184	Em^r^, *Lactobacillus–E. coli* shuttle vector	Our lab
pIB184-2179	Overexpression plasmid for *LC2W_2179*	This study
pIB184-2182	Overexpression plasmid for *LC2W_2182*	This study
pIB184-2187	Overexpression plasmid for *LC2W_2187*	This study
pIB184-2188	Overexpression plasmid for *LC2W_2188*	This study
pIB184-2189	Overexpression plasmid for *LC2W_2189*	This study


### DNA Manipulation Techniques

PCR amplifications and PCR identifications were performed using the KOD-plus-neo DNA polymerase (Toyobo, Osaka, Japan) or KOD FX polymerase (Toyobo), respectively. Thermo Fisher Scientific (United States) DNA restriction enzymes were used for cloning. Recombinant plasmids were constructed by the ClonExpress MultiS one-step cloning kit (Vazyme Biotech, Co., Ltd., Nanjing, China). Kits of Axygen Biotechnology, Co., Ltd. (Hangzhou, China) were used for extracting plasmids and chromosomal DNA.

All plasmids and oligonucleotides used in this study are listed in **Tables 1, 2**, respectively. Five genes were chosen as targets: glucose-1-phosphate thymidyltranseferase gene (*LC2W_2179*), oligosaccharide repeat unit transporter (*LC2W_2182*), hypothetical gene (*LC2W_2187*), uncharacterized EPS biosynthesis protein (*LC2W_2188*), EPS biosynthesis protein (*LC2W_2189*). LC2WΔ2179 and LC2WΔ2189 have been constructed in the previous study (Song et al., 2017). Knockout plasmids for other three genes (pLCNICK-2182, pLCNICK-2187, and pLCNICK-2188) were constructed by the one-step cloning kit, using follow DNA fragments: the backbone of plasmid pLCNICK-2179 (generated by double digestion by *Xba*I and *Apa*I), single guide RNA and homologous arms (Has) cassette (amplified by PCR using corresponding primers). To identify positive clones, colony PCR assays were conducted using a pair of primers for each mutant: the upstream primer of upstream Ha and the downstream primer of downstream Ha.

**Table 2:** Oligonucleotides used in this study.

Oligos	Sequence (5′→3′)
2182-1-upN	ACGGATCACATCTTTTTCTAAACTAGGGCCCCTTAACAACTTCTCTAGGTA
2182-1-downN	TAGGTGAACTTAAGGGGGAT
2182-2-upN	TTGGAATCCCCCTTAAGTTCACCTACATAAAAATTCTCCAATACC
2182-2-downN	GCACCGAGTCGGTGCTTTTTTTGAGTTAGCTTACGTTGGCAGAC
2182gRNAN	AAGAAAGGATGATATCACCTCTAGAAAAATAACTGAAACTGTATAGTTTTAGAGCTAGA
2187-1-up	ACGGATCACATCTTTTTCTAAACTAGGGCCCTAATTCAGAGACGTCCAAAG
2187-1-down	TAAGATTCTCTTTCTTCATGC
2187-2-up	ACCTGCATGAAGAAAGAGAATCTTACACGATAGATTCCCCCAA
2187-2-down	GCACCGAGTCGGTGCTTTTTTTGAGAATCTGAAAATGAAGAGTCAAAAG
2187gRNA	AAGAAAGGATGATATCACCTCTAGAGCTTTGGCAGGTCCTATATTGTTTTAGAGCTAGA
2188-Ha1-upN	ACGGATCACATCTTTTTCTAAACTAGGGCCCGATAAAAGGTCCTTGTCATAC
2188-Ha1-downN	TAGTAGCTTGTTTTTGAATGC
2188-Ha2-upN	TATCCGCATTCAAAAACAAGCTACTACATTTCTATTTTCTCCTTC
2188-Ha2-downN	GCACCGAGTCGGTGCTTTTTTTGAGGTCCGTATTTTAGTTAGTTT
2188gRNAN	AAGAAAGGATGATATCACCTCTAGATTTGAATTTAAAAGTTCTGAGTTTTAGAGCTAGA
2179-pIB184-up	GAAAAATATGAATGACAATGATGTTGGATCCATGCCAGATTTAGCACAG
2179-pIB184-down	GCTTATCGATAGATCTCGAGCTCTAGAATTCTTATTTGGCCAATTGCAA
2182-pIB184-up	GAAAAATATGAATGACAATGATGTTGGATCCATGAAAGTTATCAAAAACTTTATT
2182-pIB184-down	GCTTATCGATAGATCTCGAGCTCTAGAATTCCTATATTCGATTACGAAAGCG
2187-pIB184-up	GAAAAATATGAATGACAATGATGTTGGATCCGTGACAGAGAAGCGTAAG
2187-pIB184-down	AGCTTATCGATAGATCTCGAGCTCTAGAATTCTTATATTTTTCCATCGATAAATTG
2188-pIB184-up	GAAAAATATGAATGACAATGATGTTGGATCCATGAGCTTGAATGGGATT
2188-pIB184-down	GCTTATCGATAGATCTCGAGCTCTAGAATTCCTAACGCTCATCGTTACT
2189-pIB184-up	TGAAAAATATGAATGACAATGATGTTGGATCCATGAACGAGCAAATCGACC
2189-pIB184-down	GCTTATCGATAGATCTCGAGCTCTAGAATTCTCAAATCCGGCGACGGCT
2182yz-up	CGGATGAATCACAACCAT
2182yz-down	TACGCACCTTTGGTGACT
2187yz-up	CAATCAGGTCGAGGTATAC
2187yz-down	AACCTATCGCCGGTTTCA
2188yz-up	CGACATTTGATCCACTCT
2188yz-down	TTAGGGTCATTCTGACGG


To construct overexpression plasmids corresponding to the five target genes, pIB184 was used as the vector with a strong constitutive promoter P_23_ (Biswas et al., 2008). Plasmid pIB184 was double digested by *BamH*I and *EcoR*I. Subsequently, the DNA fragments *LC2W_2179, LC2W_2182, LC2W_2187, LC2W_2188, LC2W_2189* (obtained by PCR using corresponding primers) were ligated into the backbone of pIB184 (*BamH*I and *EcoR*I), respectively, to generate overexpression plasmids (pIB184-2179, pIB184-2182, pIB184-2187, pIB184-2188, pIB184-2189). The colonies were confirmed by PCR assay, using primers amplified responding genes.

### Preparation of Cells for Electroporation

Competent cells of LC2W were prepared as follows. 2 ml of overnight culture was inoculated into 50 ml MRS with 1% glycine added (MRSG) and incubated at 37°C without shaking until the optical density at 600 nm (OD_600_) reached 0.6 to 0.8. Cells were chilled on ice for 10 min and then harvested by centrifugation at 4,000 ×*g*, 4°C for 15 min. Cells were washed twice with 30 ml ice-cold 10% glycerol in water, with centrifugation at 5000 ×*g* and 4°C for 15 min and re-suspended in 0.5 ml ice-cold 10% glycerol in water. One hundred-microliter aliquots of cells were stored at -80°C.

For each electroporation, the competent cells mixed with about 100 ng (for overexpression and complementation) or 1 μg (for knockout) plasmid DNA were transferred into a 4°C precooled 2-mm cuvette (Bio-Rad, United States). Electroporation was performed at 2 kV, 200 Ω and 25 μF, using a Bio-Rad GenePulser Xcell. Then, 900 μl MMRS (MRS with 500 mM sucrose, 20 mM MgCl_2_, and 2 mM CaCl_2_) broth was added into the cuvette. The transformed cells were recovered at 37°C for 2–3 h. After that, the recovered cells were plated on MRS (with erythromycin) and incubated for 2–3 days.

### Identification of Transformants

The plasmids for gene knockout were delivered into LC2W competent cells by electroporation and cultivated for 48–96 h at 37°C. To identify positive knockout clones, PCR identification was conducted using the primers flanking outside Has on the chromosome. The wild strain LC2W was regarded as control.

To purify mutants and cure plasmids, recombinants LC2W containing different plasmids such as pLCNICK-2182, pLCNICK-2187, pLCNICK-2188 were streaked on MRS medium without erythromycin for about 2–3 times till pure mutants were obtained and plasmids were cured.

To obtained overexpression mutants, the overexpression plasmids were delivered into LC2W competent cells by electroporation, respectively. PCR identification was performed using the primers Em-up/down to identify positive overexpression clones.

The competent cells of gene knockout mutants were prepared and overexpression plasmids were delivered into the responding competent cells to obtained gene complementation clones. To identify positive complementation clones, colony PCR was conducted using the primers, which were used to amply *LC2W_2179, LC2W_2182, LC2W_2187, LC2W_2188, LC2W_2189*, respectively.

### Evaluation of EPS Production

Fermentation was carried out to analyze the EPS production of gene-knockout mutants, gene-overexpression mutants, gene-complementation mutants, and wild-type strain. 2 ml of overnight culture was inoculated into 50 ml MRS broth and incubated at 37°C without shaking for 24 h. The fermentation broth was heated in boiling water for 10 min to inactivate enzymes, and then cooled down to room temperature. Cells and coagulated proteins were removed by centrifugation (10,000 ×*g*, 4°C, 20 min). EPS was precipitated from the supernatant by adding three times volume of cold ethanol and then stored at 4°C for 24 h. After centrifugation (10,000 ×*g*, 4°C, 20 min), the precipitate was re-suspended in ultrapure water and dialyzed against water for 72 h (molecular size cut-off: 12,000–14,000 Da). The EPS was evaluated as previously described (Lokman et al., 1997).

### Statistical Analysis

All statistical analysis was performed using SPSS software. All data was initially assessed using a one-way ANOVA. All statistically different group are defined as ^∗^*P* ≤ 0.05.

## Results

### *In silico* Analysis of EPS Gene Cluster in LC2W

**FIGURE 1:**
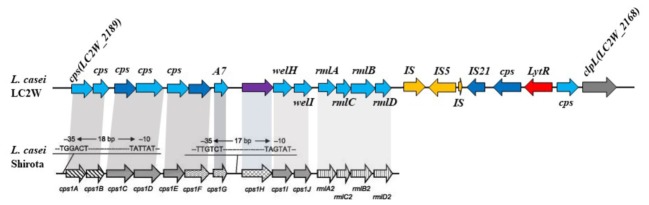
The comparative analysis of EPS gene cluster between LC2W and *Lactobacillus casei* Shirota.

In this study, gene cluster which correlated to EPS biosynthesis was hunted down based on surveying the genome sequence of LC2W (Chen et al., 2011). The putative function of each gene was listed in **Table 3**. Yasuda et al. (2008) reported a unique cluster, which involved in biosynthesis of EPS in *L. casei* Shirota (Yasuda). The hypothesis EPS cluster from *L. casei* LC2W was compared with the unique cluster from *L. casei* Shirota (**Figure 1**). The cluster of genes from *L. casei* Shirota, which have been named *cps1A, cps1B, cps1C, cps1D, cps1E, cps1F, cps1G, cps1J*, determined the synthesis of the EPS. *LC2W_2189* and *LC2W_2188* were similar to *cps1A, cps1B* in amino acid sequences, as well as *LC2W_2182* was similar to *cps1H, and LC2W_2179 was similar to rmlA2*. Due to the similarities of the EPS biosynthesis cluster from *L. casei* Shirota, *LC2W_2188* and *LC2W_2189* were speculated as EPS biosynthesis protein. In addition, *LC2W_2179* was predicted as glucose-1-phosphate thymodyltransferase protein and *LC2W_2182* was assumed as oligosaccharide repeat unit transporter protein. All genes mentioned above could regulate the production of EPS according to the blast result. Five genes were chosen to study: *LC2W_2179*, *LC2W_2182*, *LC2W_2187*, *LC2W_2188*, and *LC2W_2189*. Four of them were considered involving in EPS biosynthesis. Besides, one of hypothetical genes in the EPS gene cluster (*LC2W_2187*) was selected for further study because of its unclear characterization in EPS biosynthesis.

**Table 3:** Molecular characterization of EPS gene cluster in LC2W.

Open reading frame	Product
*LC2W_2169*	Capsular polysaccharide biosynthesis protein
*LC2W_2170*	Transcription regulator
*LC2W_2171, LC2W_2184, LC2W_2187*	Hypothetical protein
*LC2W_2172, LC2W_2173, LC2W_2174, LC2W_2175*	Transposase
*LC2W_2176*	dTDP-4-dehydrorhamnose reductase
*LC2W_2177*	dTDP-glucose 4,6-dehydratase
*LC2W_2178*	dTDP-4-dehydrorhamnose 3,5-epimerase
*LC2W_2179*)	Glucose-1-phosphate thymidyltransferase
*LC2W_2180, LC2W_2181, LC2W_2183, LC2W_2186*	Glycosyl transferase
*LC2W_2182*	Oligosaccharide repeat unit transporter
*LC2W_2185*	UDP-glucose 4-epimerase
*LC2W_2188*	Uncharacterized exopolysaccharide biosynthesis protein
*LC2W_2189*	Exopolysaccharide biosynthesis protein


### Construction of a Series of Plasmids and Electroporation

To identify the function of the genes we selected, our previously developed Crispr-Cas9^D10A^ Nickase-assisted genome editing method (Song et al., 2017) was used for gene knockout in *L. casei* LC2W. A series of plasmids were constructed to develop the gene knockout mutants of target genes (*LC2W_2182*, *LC2W_2187*, and *LC2W_2188*). The positive colonies were confirmed by a 2-kb PCR product. The result of the double digestion by *Xba*I and *Apa*I revealed that the cells contained both 12 kb of pLCNICK backbone and 2 kb of Has-sgRNA fragment (**Supplementary Figure S1**), indicating that the Has-sgRNA had been successfully incorporated into pLCNICK. Sequencing of the plasmids confirmed recombination fragments as expected. The recombination plasmids were named as pLCNICK-2182, pLCNICK-2187, and pLCNICK-2188.

The overexpression plasmids of different genes were constructed to develop the gene overexpression mutants and gene complementation mutants. The positive colonies were confirmed by a 1/1.5 kb PCR product. The result of the double digestion revealed that the cells contained both ∼6 kb pIB184 backbone and the ∼1/1.5 kb gene fragment, indicating that the target gene had been successfully incorporated into pIB184 (**Supplementary Figure S2**). These overexpression plasmids were named as pIB184-2179, pIB184-2182, pIB184-2187, pIB184-2188, and pIB184-2189.

### Construction of Gene Knockout, Gene Overexpression, and Gene Complementation Mutants of Target Genes

**FIGURE 2:**
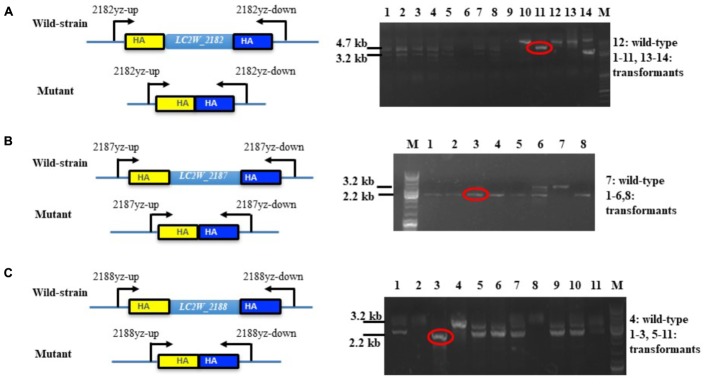
PCR identification of the gene-knockout mutants. **(A)** Identification of LC2WΔ2182 mutants. Lane 12 is the wild-type LC2W and others are transformants. The wild-type strain was used as a control. DNA fragments of 4.7 and 3.2 kb correspond to the wild-type and the deletion mutants. **(B)** Identification of LC2WΔ2187 mutants. Lane 7 is the wild-type LC2W and others are transformants. Lane M, molecular size marker. DNA fragments of 3.2 and 2.2 kb correspond to the wild-type and the deletion mutants. **(C)** Identification of LC2WΔ2188 mutants. Lane 4 is the wild-type LC2W and others are transformants. DNA fragments of 3.2 and 2.2 kb correspond to the wild-type and the deletion mutant.

The constructed gene knockout plasmids were delivered into LC2W and the gene knockout mutants of *LC2W_2187* or *LC2W_2188* were confirmed by a 2.2 kb PCR product, which was 1 kb smaller than the wild-type LC2W. Gene knockout mutant of *LC2W_2182* was evidenced by a 3.2 kb PCR product, which was 1.5 kb smaller than LC2W (**Figure 2**). The difference existed between DNA fragments amplified by mutants and wild-type strain consisted with the length of target genes. It implied that the gene knockout mutants (LC2WΔ2182, LC2WΔ2187, and LC2WΔ2188) were constructed successfully using the Crispr-Cas9^D10A^ Nickase-assisted genome editing plasmid (pLCNICK).

The EPS production between overexpression mutant, complementation mutants and wild-type strain should compare to instruct the result of gene knockout further. Gene overexpression plasmids were transferred into competent cells of LC2W. The result of colony PCR assays showed that 700 bp fragments were obtained from *LC2W_2179, LC2W_2182, LC2W_2187, LC2W_2188*, and *LC2W_2189* overexpression transformants. The DNA fragments amplified from transformants were consistent with the size of erythromycin gene. It is shown that gene overexpression mutants (LC2W pIB184-2179, LC2W pIB184-2182, LC2W pIB184-2187, LC2W pIB184-2188, LC2W pIB184-2189) were constructed successfully using the corresponding plasmids.

Construction of gene complementation mutants was the same as above, only the competent cells of the corresponding gene knockout mutants were used. The gene complementation mutants were named as LC2WΔ2179 pIB184-2179, LC2WΔ2182 pIB184-2182, LC2WΔ2187 pIB184-2187, LC2WΔ2188 pIB184-2188, and LC2WΔ2189 pIB184-2189.

### Production of EPS by Mutants and Wild-Type

**FIGURE 3:**
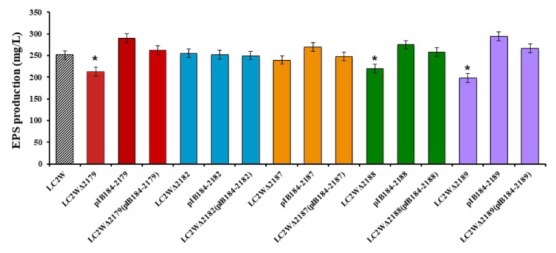
EPS (exopolysaccharide) production of gene knockout mutants, gene overexpression mutants and gene complementation mutants. The EPS production of wild-type LC2W was used as a control. Asterisk denotes statistical significance (*P* ≤ 0.05) to control.

Since the chosen target genes were speculated as the key factors in EPS biosynthesis, the EPS production of mutants and wild strain was compared. As shown in **Figure 3**, the gene knockout mutants LC2WΔ2179, LC2WΔ2188, and LC2WΔ2189 yield fewer EPS compared with LC2W. EPS production decreased 15, 13, and 21% compared with the wild-type strain, respectively (^∗^*P* ≤ 0.05). The improvement of EPS production of the corresponding gene overexpression mutants (LC2W pIB184-2179, LC2W pIB184-2188, and LC2W pIB184-2189) confirmed the result above. EPS production of these gene overexpression mutants increased 16, 10, and 18% compared with the wild-type strain. The same as LC2WΔ2189 (Song et al., 2017), gene knockout mutant LC2WΔ2188 showed reduced adhesion compared to that of LC2W when cultivated on MRS plate. Moreover, the mutant LC2WΔ2188 had a tendency to coagulate and precipitate in liquid MRS medium. This tendency to coagulate and the precipitate was disappeared in the gene complementation mutant LC2WΔ2188 pIB184-2188 and LC2WΔ2189 pIB184-2189.

There was no significant difference of the EPS production between other gene knockout mutants (LC2WΔ2182 and LC2WΔ2187) and the wild-type strain. These two genes (*LC2W_2182* and *LC2W_2187*) were considered not to be involved in the EPS biosynthesis. The same result of the comparison between the corresponding gene overexpression mutants and wild-type strain was consistent with this conclusion (**Figure 3**).

## Discussion

Exopolysaccharides produced by LAB, are generally recognized as safe (GRAS) food additives. It was reported that EPSs possess beneficial effects on health such as a decrease of blood cholesterol and immune-stimulatory capacities (Hosono et al., 1997; Bleau et al., 2010; Starovoitova et al., 2012). Because of their safe and healthy typical feature, EPSs have received special attention since their potential application in food industry. Some reports focused on the chemical and structural characterization of EPSs and some reviews addressed the health-promoting prebiotic effect of EPS (Salazar et al., 2016). Researchers tried to improved EPS production by genetic and metabolic engineering, but they all chose to overexpress heterogeneous genes due to the lack of efficient genetic tools in LAB. Since an efficient Crispr-Cas9^D10A^ Nickase-assisted genome editing method in *L. casei* was established (Song et al., 2017), we tried to study the relationship between *eps* genes and EPS production and regulated EPS production by genetic engineering using the homologous genes.

Yasuda et al. (2008) reported that some genes from a unique cluster were involved in biosynthesis of EPS in *L. casei* Shirota. The proteins encoded by genes from this unique cluster were predicted and they had similarities to various extents to known proteins involved in biosynthesis of EPS from other LAB (Yasuda et al., 2008). In this study, we have genetically identified a cluster of genes whose products play an important role in EPS biosynthesis in genome of *L. casei* LC2W. The genome sequence of *L. casei* LC2W was blasted with *L. casei* Shirota and a similar cluster was found. Because of the similarity between these two EPS biosynthesis clusters, functions of some genes from the hypothesis cluster were predicted.

To investigate the roles of these genes in EPS production, gene knockout mutants, gene overexpression mutants and gene complementation mutants developed from those five genes were constructed. EPS production of the series mutants and wild-type strain were measured and compared. From the result, EPS production of LC2WΔ2179, LC2WΔ2188, and LC2WΔ2189 decreased compared with the wild-type strain respectively. Corresponding gene overexpression mutants (LC2W pIB184-2179, LC2W pIB184-2188, LC2W pIB184-2189) produce more EPS compared with the wild strain. The EPS production of the complementation mutant LC2WΔ2179 pIB184-2179 was similar with the wild-type strain, the same trend was observed in LC2WΔ2188 pIB184-2188 and LC2WΔ2189 pIB184-2189, which confirmed *LC2W_2179*, *LC2W_2188*, and *LC2W_2189* were critical in EPS biosynthesis.

*LC2W_2188* and *LC2W_2189* were assumed as capsular polysaccharide biosynthesis genes, and *LC2W_2179* was predicted as nucleotide sugar substrate synthesis gene. The above three genes played important roles in EPS biosynthesis.

Gene knockout mutants LC2WΔ2188 and LC2WΔ2189 showed a tendency to coagulate and precipitate compared to LC2W when cultivated in liquid MRS medium. For this result, we proposed that *LC2W_2188* and *LC2W_2189* played an important role in EPS biosynthesis. Lack of either of them could cause downtrend of EPS production. They were speculated to cause some changes in the constitution or structure of the EPS, which has been demonstrated to be involved in stabilizing LAB growth (Ruas-Madiedo and de los Reyes-Gavilan, 2005). EPSs are extensively used to improve stability, rheological properties, and texture of food products in food industry because of its hydrocolloid properties (Juvonen et al., 2015). We purposed that lack of *LC2W_2188* or *LC2W_2189* caused some changes of EPS and decreased its hydrocolloid properties in liquid medium.

Although *LC2W_2182* and *LC2W_2187* are members of this EPS biosysthesis cluster, their contribution to the EPS production was not apparent. The change of EPS production was not observed in the gene knockout strains (LC2WΔ2182 and LC2WΔ2187) and the corresponding gene overexpression mutants. It implied that *LC2W_2182* and *LC2W_2187* were not involved in EPS biosynthesis in *L. casei* LC2W. Further analysis of the function of these genes is needed to clarify the role of each gene product in EPS biosynthesis.

## Conclusion

Thirteen mutants related to the five target genes were constructed and evaluated EPS yield. The result showed that *LC2W_2179, LC2W_2188*, and *LC2W_2189* were important in EPS biosynthesis while *LC2W_2182* and *LC2W_2187* were not involved in this process. At the same time, *LC2W_2188* and *LC2W_2189* were related to the hydrocolloid properties of LC2W.

## Author Contributions

XS performed the experiments and wrote the manuscript. ZX blasted the genes. LK assisted the statistical analysis. GW and LA designed the experiments.

## Conflict of Interest Statement

The authors declare that the research was conducted in the absence of any commercial or financial relationships that could be construed as a potential conflict of interest.

## Supplementary Material

FIGURE S1 Identification of the plasmids for gene-knockout mutants. Lanes 1, 2 were the result of colony PCR assays. PCR-amplified fragments 2 kb correspond to Has-sgRNA cassette. Lanes 3–6 were the result of double digestion. Fragments 2 and 12 kb correspond to Has-sgRNA cassette for target genes and the backbone of pLCNICK. Lane M, molecular size marker.

FIGURE S2 Identification of the plasmids for gene-overexpression and gene-complementation mutants. **(A)** Lanes 1–3, 4–6, 7–9, 10–12 were the result of colony PCR assays for the target genes *LC2W_2179, LC2W_2187, LC2W_2188, LC2W_2189*. Lane M, molecular size marker. **(B)** Lanes 1–2 were the result of colony PCR assays for *LC2W_2182.* **(C)** The result of double digestion. Fragments 1 and 6 kb correspond to gene fragment and the backbone of pIB184.
