# Supplementary figures and images for: Relationship Between Putative eps Genes and Production of Exopolysaccharide in Lactobacillus casei LC2W
Source: Front Microbiol. 2018 Aug 17;9:1882. doi: 10.3389/fmicb.2018.01882 (PMC6107683; doi:10.3389/fmicb.2018.01882)

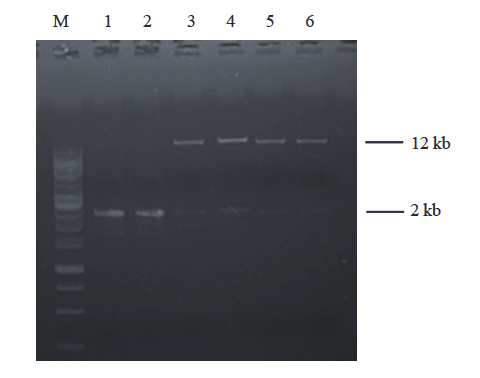

Supplement: FIGURE S1 [file Image_1.PNG]

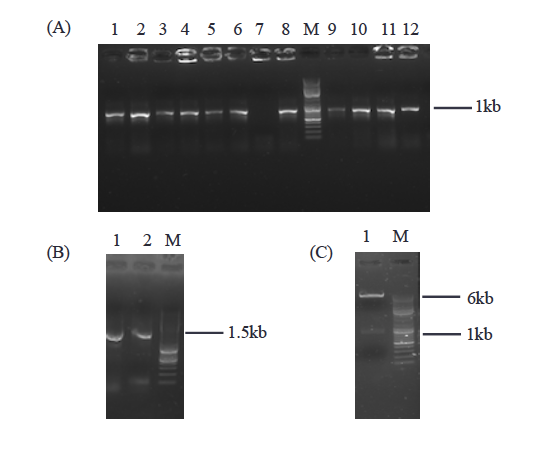

Supplement: FIGURE S2 [file Image_2.PNG]
